# Estimating the Prevalence of GNE Myopathy Using Population Genetic Databases

**DOI:** 10.1155/2024/7377504

**Published:** 2024-08-29

**Authors:** Alexa Derksen, Rachel Thompson, Madeeha Shaikh, Sally Spendiff, Theodore J. Perkins, Hanns Lochmüller

**Affiliations:** ^1^ Department of Cellular and Molecular Medicine University of Ottawa, Ottawa, Ontario, Canada; ^2^ Children's Hospital of Eastern Ontario Research Institute, Ottawa, Ontario, Canada; ^3^ Translational and Molecular Medicine University of Ottawa, Ottawa, Ontario, Canada; ^4^ Regenerative Medicine Program Ottawa Hospital Research Institute, Ottawa, Ontario, Canada; ^5^ Ottawa Institute of Systems Biology Department of Biochemistry Microbiology and Immunology University of Ottawa, Ottawa, Ontario, Canada; ^6^ Brain and Mind Research Institute University of Ottawa, Ottawa, Ontario, Canada; ^7^ Division of Neurology Department of Medicine The Ottawa Hospital, Ottawa, Ontario, Canada; ^8^ Department of Neuropediatrics and Muscle Disorders Faculty of Medicine Medical Center – University of Freiburg, Freiburg, Germany; ^9^ Centro Nacional de Análisis Genómico (CNAG) Barcelona Institute of Science and Technology (BIST), Barcelona, Spain

**Keywords:** genetic database, GNE myopathy, neuromuscular disease, population, prevalence, rare disease, variants

## Abstract

GNE myopathy (GNEM) is a rare autosomal recessive disorder characterized by progressive skeletal muscle wasting starting in early adulthood. The prevalence of GNEM is estimated to range between one and nine cases per million individuals, but the accuracy of these estimates is limited by underdiagnosis, misdiagnosis, and bias introduced by founder allele frequencies. As GNEM is a recessive disorder, unaffected carriers of single damaging variants can be expected to be found in the healthy population, providing an alternative method for estimating prevalence. We aim to estimate the prevalence of GNEM using allele frequencies obtained from healthy population genetic databases. We performed a review to establish a complete list of all known pathogenic GNEM variants from both literature and variant databases. We then developed standardized filtering steps using in silico tools to predict the pathogenicity of unreported *GNE* variants of uncertain clinical significance and validated our pathogenicity inferences using Mendelian Approach to Variant Effect pRedICtion built in Keras (MAVERICK) and AlphaMissense. We calculated conservative and liberal disease prevalence estimates using allele frequencies from the Genome Aggregation Database (gnomAD) population database by employing methodologies based on the assumptions of the Hardy–Weinberg Equilibrium. We additionally calculated estimates for disease prevalence removing the contribution of unique variant combinations that either do not cause myopathy in humans or result in embryonic lethality. We present the most comprehensive list of reported pathogenic *GNE* variants to date, together with additional variants predicted as pathogenic by in silico methods. We provide additional pathogenicity scores for these variants using new pathogenicity prediction tools and present a set of estimates for GNEM prevalence based on the different assumptions. Our most conservative estimate suggested a prevalence of 18.46 cases per million, while our most liberal estimate places the prevalence at 95.42 cases per million. When accounting for variant severity, this range drops to 11.00–87.68 cases per million. Our findings indicate that the true global prevalence of GNEM is greater than previous predictions underscoring that this condition is considerably more widespread than previously believed.

## 1. Introduction

GNE myopathy (GNEM) is a rare autosomal recessive disorder which usually presents in early adulthood. It is characterized by progressive skeletal muscle atrophy which begins distally and generally spares the quadriceps muscles [1, 2]. As the disease progresses and more muscle groups become involved, patients are at increased risk of falls, experience impaired physical functioning, and become wheelchair dependent and ultimately reliant on caregivers. The underlying genetic cause of GNEM is linked to the *GNE* gene located on Chromosome 9, which produces a bifunctional enzyme known as UDP-N-acetylglucosamine 2-epimerase/N-acetylmannosamine kinase [3]. This enzyme performs back-to-back steps in the biosynthesis of sialic acid (SA) [4]. While the precise mechanism of disease has yet to be elucidated, it is hypothesized that reduced sialyation of crucial muscle glycans could play a role [5].

Previous reports indicate that there are approximately 300 known GNEM-causing variants in *GNE* [6]. Interestingly, there does not seem to be a strong correlation between the location of mutations along the GNE protein and the disease severity [7]. GNEM is not the only disorder resulting from mutations in *GNE*; sialuria, an autosomal dominant disorder, characterized by excessive SA production, is caused by mutations in a specific domain of GNE which impair its negative feedback mechanism [8]. Several other disorders including isolated thrombocytopenia, pure motor neuropathy, and amyotrophic lateral sclerosis have also been linked to specific mutations in *GNE* [9–11].

There are seven well-documented founder mutations associated with GNEM found in the United Kingdom (p.Asp409Tyr and p.Ala662Val), the Middle East (p.Met743Thr), Japan (p.Asp207Val and p.Val603Leu), India (p.Val727Met), and Bulgarian Roma (p.Ile618Thr) populations [7]. Most GNEM-causing mutations are missense variants; however, nonsense, splice site, copy number variants (CNVs), and even synonymous variants have been described. The presence of two null mutations on opposite alleles is believed to be incompatible with life; this has been supported by studies in which knocking out the GNE protein in mice resulted in embryonic lethality [12, 13]. Additionally, one of the Japanese founder mutations, p.Asp207Val, is presumed to cause a very mild or no disease phenotype when it appears in homozygosity [14, 15]. Thus, the p.Asp207Val mutation generally must appear in compound heterozygosity with another more deleterious variant to be pathogenic and cause GNEM. The Indian founder mutation (p.Val727Met) is another unique variant of interest. While this variant has been reported to cause GNEM in homozygosity, its high allele frequency of 7.45e^−4^ and the reported 12 healthy homozygotes in the Genome Aggregation Database (gnomAD) v4.0.0 suggest that it is not fully penetrant in homozygosity [16]. Lastly, a common African/African American variant (p.Asp239Glu) has emerged as an additional variant of interest. While this variant has been reported to cause disease when in trans with another deleterious *GNE* variant, its high allele frequency of 6.95e^−4^ and 11 reported homozygotes in gnomAD (v4.0.0) suggest that it is unlikely to be disease-causing in homozygosity [10, 17, 18].

To ensure that health care systems can provide adequate resources and care, it is imperative to have accurate estimates of disease prevalence. Additionally, from a research perspective, accurate estimates are necessary to ensure successful design, prioritization, and implementation of clinical trials. In rare disease, prevalence estimates are typically based on epidemiological approaches and/or sparse allele frequency data. As such, they are limited by misdiagnoses and underdiagnoses and further complicated by founder mutations and consanguinity [19]. It is thus believed that these estimates are an underrepresentation of true disease prevalence. As GNEM is a recessive disorder, single variants are only pathogenic when present in homozygosity or in compound heterozygosity with another deleterious variant. Thereby, these single variants can be found in the healthy general population, as alone they are not disease-causing. This provides an opportunity for prevalence estimation, as with the availability of online large-scale population genomic databases, the prevalence of autosomal recessive disorders can be calculated based on the frequency of single variants in this healthy population. Here, we not only use the availability of population genetic databases and allele frequencies to calculate the disease prevalence for previously reported pathogenic mutations but also go beyond, creating filtering strategies to predict additional likely pathogenic variants to use in broader estimates of disease prevalence. We performed calculations according to previously published methods which utilized the Hardy–Weinberg Equilibrium as well as the more complex Bayesian methodology [19–23]. Ultimately, we achieved a comprehensive set of estimates for disease prevalence that give us a range for the true prevalence of GNEM.

## 2. Materials and Methods

### 2.1. Identification of Pathogenic Variants From Literature and Variant Databases

**Figure 1:**
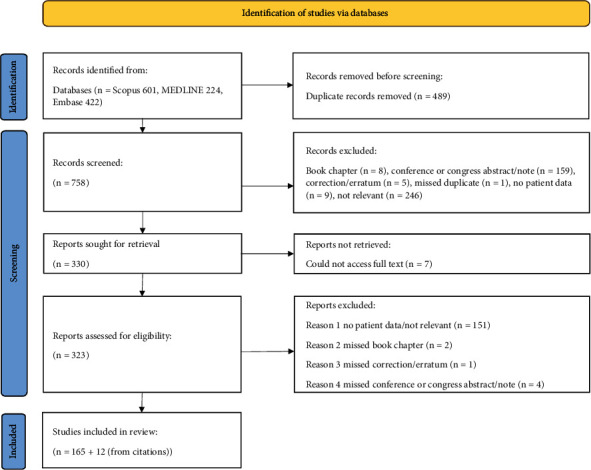
Literature review flow diagram. PRISMA flow diagram detailing the databases searched, the number of abstracts screened, the full texts retrieved, and the number of studies included.

Published pathogenic GNEM-causing variants were found through a literature search. Scopus, MEDLINE, and Embase were systemically searched (on 22/07/2022) using the following keywords: GNE gene, GNE disease, GNE and thrombocytopenia, GNE myopathy, GNE and Hereditary Inclusion Body Myopathy, GNE and Quadriceps Sparing Myopathy, GNE and Nonaka Distal Myopathy, GNE and Distal Nonaka Myopathy, UDP-N-acetylglucosamine 2-epimerase N-acetylmannosamine kinase and myopathy, GNE and Sialuria. Articles were added to a library on Zotero, and duplicates were removed. A title and abstract screen were performed, and articles that did not fit the scope of the study were removed. A full-text search was conducted in the remaining articles, and published pathogenic *GNE* variants were recorded (Figure 1). The online variant pathogenicity databases ClinVar (https://www-ncbi-nlm-nih-gov.ca/clinvar/), Leiden Open Variation Database (LOVD) (https://www.lovd.nl/), and Human Gene Mutation Database (HGMD) (https://www.hgmd.cf.ac.uk/) were also searched for *GNE* variants (on 18/07/2022), and those that were identified as likely pathogenic or pathogenic were included for analysis. Across both searches, all variants were converted to the same transcript, NM_001128227, and duplicates were removed. Furthermore, variants that had been attributed exclusively to diseases other than GNEM were removed from the final list.

### 2.2. Determination of Allele Frequencies and Identification of Additional Likely Pathogenic Variants

Allele frequencies for all *GNE* variants present in the gnomAD database (v2.1.1 and v4.0.0) were extracted. Those identified as pathogenic or likely pathogenic through the literature and database searches were assumed for the purposes of this analysis to be true pathogenic variants and were taken for direct use in the prevalence estimate calculations. All remaining *GNE* variants present in gnomAD v2.1.1 underwent additional filtering and analysis as described here to establish putative pathogenicity and develop additional variant lists for more comprehensive prevalence estimates.

**Figure 2:**
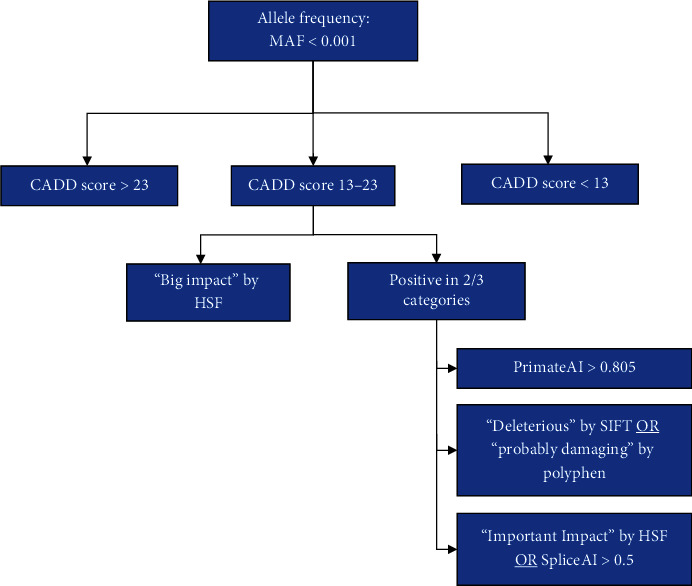
Flow chart of the filtering steps to determine the pathogenicity of GNE variants of uncertain significance. Variants of uncertain significance were first filtered by minor allele frequency (MAF) with a cut-off set at 0.001. Those variants that were under this cut-off underwent additional filtering based on their respective CADD scores. If they had a CADD score greater than 23, they were considered pathogenic, whereas if their CADD score was below 13, they were considered benign. Variants that fell in the 13–23 range underwent further filtering based on additional in silico tools. Variants that were found to have a “Big Impact” on HSF or who met two out of three cut-off criteria in (1) PrimateAI, (2) SIFT or Polyphen, or (3) HSF or SpliceAI were also considered to be likely deleterious. Abbreviations: CADD: Combined Annotation Dependent Depletion; HSF: Human Splicing Finder; SIFT: Sorting Intolerant From Tolerant; Polyphen: Polymorphism Phenotyping.

**Figure 3:**
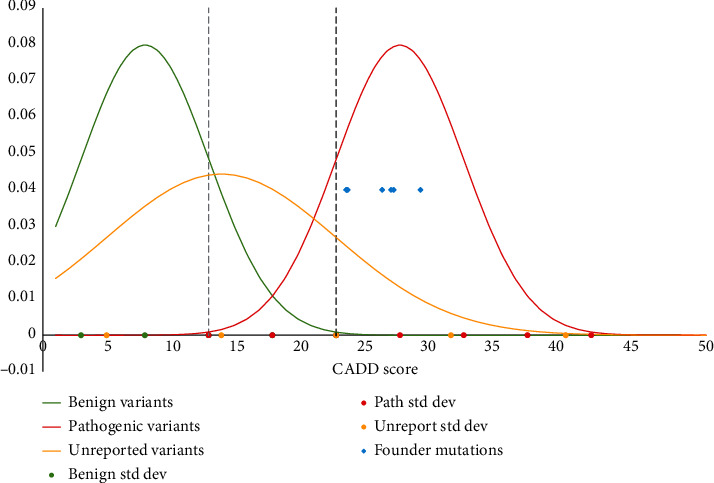
Normal distribution of CADD scores for benign, pathogenic, and unreported variants in *GNE*. Standard deviations are indicated as points on the *x*-axis. CADD scores for the GNEM founder mutations are indicated as blue-coloured diamonds. A lower cut-off for pathogenicity was set at 13 which is three standard deviations from the mean of the pathogenic curve (dashed grey line). An upper cut-off for pathogenicity was set at 23 which is three standard deviations from the mean of the benign curve (dashed black line).

All variants classified as benign or likely benign in gnomAD v2.1.1 were further investigated via literature evidence, allele frequency, and in silico pathogenicity inferences to ensure that they could truly be considered benign and were excluded from further calculations. Variants that were unclassified or classified as being of unknown clinical significance in gnomAD v2.1.1 underwent a set of filtration steps to determine which were plausibly pathogenic despite being unreported as disease-causing (Figure 2). They were filtered first by minor allele frequency (MAF) with a cut-off set at 0.001. This cut-off was chosen as it was the highest frequency in the known pathogenic variant list. Next, the normal distributions for the Combined Annotation Dependent Depletion (CADD) scores of the literature and database-extracted pathogenic variants as well as the known benign variants were plotted (Figure 3). These curves were used to determine CADD score cut-offs to use on the variants of uncertain significance. All variants with a CADD score greater than 23 were considered likely pathogenic, while those with scores below 13 were considered likely benign. These cut-offs were chosen as 23 fell three standard deviations above the mean of the benign curve, while 13 fell three standard deviations below the mean of the pathogenic curve. Variants with scores between 13 and 23 remained uncertain and were subjected to additional filtering using a suite of in silico pathogenicity prediction tools: Sorting Intolerant From Tolerant (SIFT), Polymorphism Phenotyping (Polyphen) v2, PrimateAI, SpliceAI, and Human Splicing Finder (HSF). These variants were deemed to be pathogenic if they had a “big impact” on splicing as predicted by HSF or were “positive” in two out of the three in silico categories: (1) splicing tools (HSF—important impact, SpliceAI—> 0.5), (2) SIFT (deleterious) or Polyphen (probably damaging or damaging), or (3) PrimateAI > 0.805.

This strategy resulted in four groups of variants listed here in order of decreasing confidence in pathogenicity: (1) known GNEM pathogenic variants, (2) known GNEM pathogenic variants plus variants of uncertain significance with CADD > 23, (3) known GNEM pathogenic variants plus variants of uncertain significance with CADD > 23 plus variants of uncertain significance with CADD between 13 and 23 meeting additional in silico criteria, and lastly, (4) known GNEM pathogenic variants plus all variants of uncertain significance with CADD > 13. These lists were used in various combinations to obtain estimates of disease prevalence.

### 2.3. Prevalence Estimation: Maximum Likelihood and Bayesian Estimates

Worldwide as well as population-specific prevalence estimates for GNEM were calculated using the sum of allele frequencies obtained from gnomAD under the assumption of the Hardy–Weinberg Equilibrium using the equation *p*^2^ + 2*pq* + *q*^2^ = 1  [22, 23]. Here, *q* = *Σ*_*i*_*q*_*i*_^ml^ is equal to the sum of the individual allele frequencies, estimated by maximum likelihood as the ratio of the allele count in gnomAD divided by the total allele number observed at the variant's position. The *q*^2^ term is then the frequency of the disease-causing genotype. We have termed this initial calculation the “maximum likelihood” approach.

Since the Hardy–Weinberg equation does not account for the inaccuracies introduced by the limited size of population databases, which, for ultrarare variants are not truly representative of the global population, we also chose to perform two additional calculations using a Bayesian methodology. Our methodology made use of open-source code published by Liu et al. [19] and Lake et al. [21] which was further developed for the different scenarios described in this Materials and Methods section. The Bayesian approach calculates belief distributions over possible true allele frequencies, based in part on the knowledge of typical frequencies of different variant types [19], and on the observed allele counts in gnomAD. In our first Bayesian approach, each allele frequency above is replaced by its expectation under the posterior distribution, *E*(*q*_*i*_^bay^), resulting in the prevalence estimate *q*^2^ = (*Σ*_*i*_ *E*(*q*_*i*_^bay^))^2^. In our second Bayesian approach, we take the expectation of *q*^2^ with respect to the Bayesian beliefs, which generates the prevalence estimate *E*(*q*^2^) = Var(*q*) + (*E*(*q*))^2^ = *Σ*_*i*_ Var(*q*_*i*_^bay^) + (*Σ*_*i*_ *E*(*q*_*i*_^bay^))^2^ (https://github.com/theodorejperkins/Monogenic_Prevalence_with_Severities; Supporting Information 1).

We performed our three calculations on our four different sets of variants to establish conservative and liberal prevalence estimates. We performed estimates using data from gnomAD version 4.0.0 on our list of previously known pathogenic GNEM variants.

### 2.4. Prevalence Estimation: Refinements to Account for Variant-Specific Effects

As a second step, we updated our methodology to prevent certain variant combinations from being considered as they would not result in living individuals with the disease, either because they are so severe that they result in an embryonically lethal phenotype or so mild that they do not result in disease. We removed the combination of two null variants appearing together as this would result in embryonic lethality. We also removed the combination of the Japanese founder mutation p.Asp207Val, the Indian founder mutation p.Val727Met, and the common African/African American variant p.Asp239Glu from appearing in homozygosity. The Japanese p.Asp207Val founder mutation appears to be mild and generally requires the presence of another more deleterious variant in compound heterozygosity to be disease-causing [14]. While a handful of individuals with this variant in homozygosity have been reported as presenting with GNEM, it is largely believed that most homozygotes do not develop any apparent disease [15, 24–26]. The Indian founder mutation has been reported to cause disease in homozygosity [16]; however, this mutation appears at a very high allele frequency in the healthy population, and gnomAD (v4.0.0) reports 12 healthy individuals with this variant in homozygosity, supporting the notion that it is likely not fully penetrant. Similarly, the common African/African American variant p.Asp239Glu has a high allele frequency in the healthy population, and gnomAD (v4.0.0) reports 11 healthy individuals with this variant in homozygosity. This variant was initially reported as a single nucleotide polymorphism [27]; however, a more recent study has demonstrated a splicing pathomechanism [17]. It is therefore proposed that this variant does not cause disease in homozygosity but rather requires the presence of another severe *GNE* variant in trans to cause disease [14]. Lastly, we removed the possibility of synonymous variants occurring in homozygosity from being incorporated into the estimate of disease prevalence as these are unlikely to be disease-causing. Supporting Information 1 contains details of the general calculation accounting for different levels of variant severity.

### 2.5. Validating Variants and Filtering Strategy Against Novel Artificial Intelligence (AI) Tools: MAVERICK (a Mendelian Approach to Variant Effect pRedICtion built in Keras) and AlphaMissense

While our methods for selecting additional potentially deleterious variants that are unreported as disease-causing were designed to be as robust and reproducible, our approach of including these variants in our more liberal estimates of prevalence is of necessity speculative owing to lack of real-world evidence of their pathogenicity. As an additional step to validate our filtering approach while also testing the utility of novel AI variant prediction tools, we determined variant pathogenicity scores MAVERICK [28] and AlphaMissense [29]. We downloaded the precomputed MAVERICK scores for all possible autosomal missense mutations from the respective GitHub page (https://github.com/ZuchnerLab/Maverick). Similarly, we downloaded the precomputed AlphaMissense scores for all possible human amino acid substitutions and missense variants from their respective Github page (https://github.com/google-deepmind/alphamissense). We next extracted the scores for variants across the *GNE* gene. This list was cross-referenced against our list of database and literature-known pathogenic GNEM variants. The respective MAVERICK and AlphaMissense scores for these variants were plotted across the protein sequence. We also extracted the MAVERICK scores for our three additional lists of variants of unknown clinical significance and plotted these again across the protein sequence.

## 3. Results

### 3.1. Literature and Database Search

**Figure 4:**
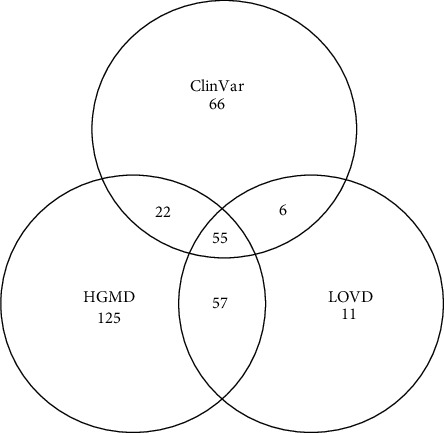
Venn diagram of *GNE* pathogenic variants from database searches. ClinVar is the National Institutes of Health human variations and phenotype database, LOVD is the Leiden Open Variation Database, and HGMD is the Human Gene Mutation Database. A total of 342 *GNE* variants were found in these databases.

**Figure 5:**
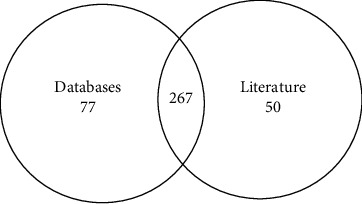
Venn diagram of *GNE* pathogenic variants from literature and database searches. A total of 394 *GNE* variants were found between the database and literature searches.

A literature search was conducted across multiple databases and resulted in the retrieval of 601 articles from Scopus, 224 from MEDLINE, and 422 from Embase. After identifying and removing duplicate records, a total of 758 distinct articles remained. These articles were subjected to screening, with 330 advancing to full-text retrieval. Ultimately, 165 articles provided information on causative *GNE* variants and were included in this review. In addition, 12 more articles were obtained from citations within the 165 papers (Figure 1). This comprehensive search resulted in a total of 2842 variants. Upon converting these variants to a single transcript (NM_001128227) and eliminating duplicates, 317 unique *GNE* variants remained. A database search conducted in parallel on ClinVar, HGMD, and LOVD yielded 149, 259, and 129 variants, respectively (Figure 4). An additional three variants were added from our in-house patient database. After careful removal of duplicates, the database search yielded a total of 345 *GNE* variants. These results in combination with those from the literature search yielded a total count of 394 pathogenic *GNE* variants (Figure 5). However, as previously mentioned, GNEM is not the only disease caused by mutations in the *GNE* gene. As such, we excluded 18 of the 394 variants as they have been associated with conditions other than GNEM, specifically sialuria, isolated thrombocytopenia, motor neuropathy, and amyotrophic lateral sclerosis. This action resulted in a refined set of 376 variants linked to GNEM (Supporting Information 2).

### 3.2. MAVERICK and AlphaMissense Predictions

**Figure 6:**
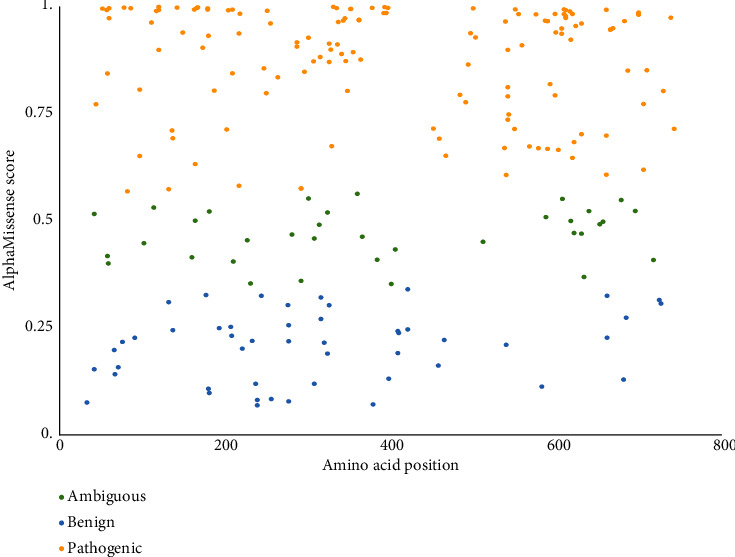
AlphaMissense predictions for known pathogenic GNEM variants. For each database and literature-reported pathogenic GNEM variant, a numerical score between 0 and 1 was assigned. Pathogenic > 0.8 shown in yellow, ambiguous 0.4–0.8 shown in green, and benign < 0.4 shown in blue.

We compared the list of variants obtained through our filtering steps to the novel AI tools MAVERICK and AlphaMissense which assign pathogenicity scores to missense and, in the case of MAVERICK, also nonsense variants [28, 29]. Both tools assign scores on a scale of 0 to 1 with 1 being the strongest. We found that AlphaMissense performed poorly in its predictions of the pathogenicity of our literature and database-reported pathogenic GNEM variants, the variants with the strongest real-world evidence of pathogenicity. Of the 223 known pathogenic GNEM variants for which AlphaMissense provided pathogenicity scores, only 139 were predicted to be pathogenic by AlphaMissense (Figure 6). Of the remaining variants, 35 were predicted to be ambiguous and 49 were benign. The variability of these results aligns with those found in a recent publication which tested the utility of AlphaMissense in classifying *CFTR* variants and emphasizes the need for caution especially when relying on AlphaMissense predictions for variants that do not have a strong effect on the protein [30]. On a broader scale, there are several limitations to the AlphaFold-derived system on which AlphaMissense is in part trained, most notably its weak correlation in predicting the impact of single mutations on protein function and stability [31].

**Figure 7:**
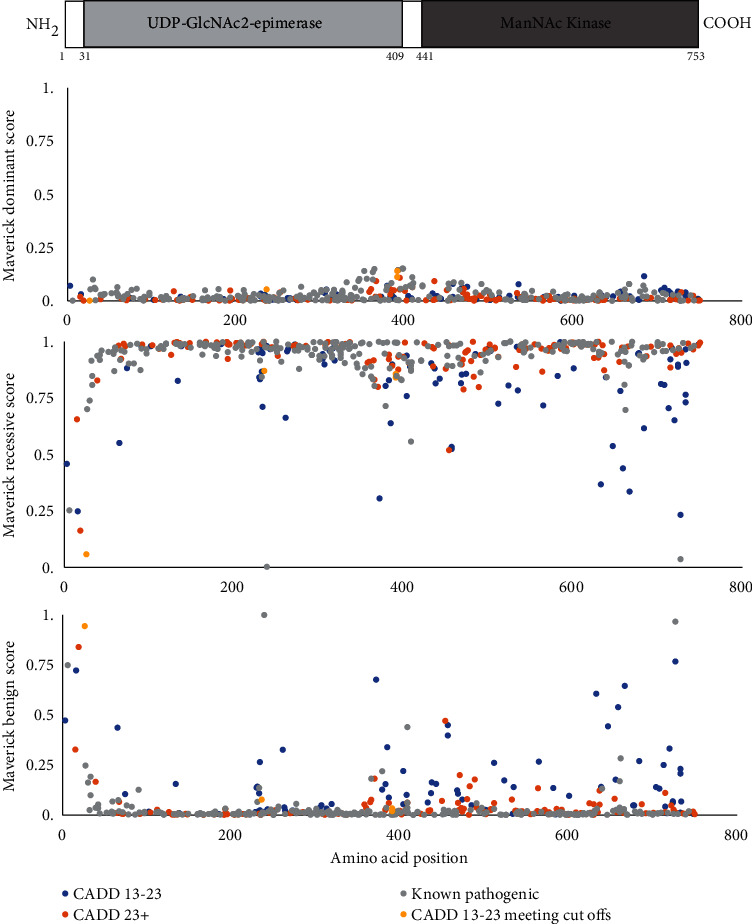
MAVERICK predictions for variants in GNE. For each variant, the MAVERICK-predicted dominant, recessive, and benign scores are shown. A diagram of the domains of the GNE protein is shown at the top. Known pathogenic variants are in grey, uncertain variants with a CADD score > 23 are shown in orange, uncertain variants with a CADD score between 13 and 23 but meeting the additional in silico criteria are shown in yellow, and the remaining uncertain variants with a CADD score between 13 and 23 are shown in blue.

Predictions obtained from MAVERICK aligned more closely with real-world evidence, with only three of the 261 known pathogenic variants with MAVERICK-associated scores not being predicted to be pathogenic in recessive inheritance (Figure 7). These three variants are c.18T>A (p.Tyr6^∗^), c.717T>G (p.Asp239Glu), and c.2179G>A (p.Val727Met) which have associated allele frequencies (gnomAD v2.1.1) of 0.000182, 0.001161, and 0.001492, respectively, the largest of all our known pathogenic GNEM variants. The p.Tyr66^∗^ variant has been reported by two clinical labs on ClinVar as being pathogenic/likely pathogenic. In contrast, p.Asp239Glu, the common African/African American variant, has been reported in the literature in two patients with GNEM and one with a motor axonal neuropathy, with functional validation demonstrating its impact on splicing [10, 17, 18]. The final variant that was predicted to be benign by MAVERICK, p.Val727Met, is the well-established GNEM Indian founder mutation. As MAVERICK is trained using allele frequency data, this may account for the benign predictions of these three high-allele-frequency GNEM-causing variants [28]. A caveat regarding the success of MAVERICK in predicting known pathogenic variants to be pathogenic is that variants from ClinVar were also part of the tool's training data [28], so it can be assumed that any *GNE* variant that was present in the ClinVar training dataset should automatically be correctly predicted by the tool. Nevertheless, MAVERICK also correctly predicted pathogenicity of several known pathogenic variants that were not present in ClinVar. Examples of such variants include p.Val653Gly, p.Gly395Arg, and p.Gly700Arg which had associated MAVERICK scores of 0.94, 0.93, and 0.97, respectively. As a result of these findings, we considered MAVERICK to be a more accurate tool for predicting the pathogenicity of GNEM variants and used it to assess our variants of uncertain significance (Figure 7).

### 3.3. Calculation 1: Disease Prevalence Including Only Known Pathogenic GNEM Variants

All variants in our known pathogenic variant list were cross-referenced with the *GNE* variants present in the gnomAD population database (version 2.1.1 and 4.0.0). Only 97 of the 376 variants were found to be present in gnomAD version 2.1.1, while 155 were present in gnomAD version 4.0.0 (Supporting Information 2 and 4). The remaining variants were presumably too rare given the size of the gnomAD sample (version 2.1.1: 125,748 exomes and 15,708 genomes, version 4.0.0: 730,947 exomes and 76,215 genomes) or belonged to populations that are underrepresented in gnomAD. The sum of the allele frequencies of the reported GNEM-causing variants alone was used to calculate our most conservative disease prevalence estimate. Based on the maximum likelihood estimate, the prevalence of GNEM worldwide is estimated to be 18.46 per million individuals (gnomAD v2.1.1) and 11.90 per million individuals (gnomAD v4.0.0) (Table 1). This estimate can also be broken down by ancestry group based on the data available in gnomAD. We found that the South Asian ancestry group had the highest prevalence in gnomAD version 2.1.1 with an estimated prevalence of 201.88 individuals per million while the European (Finnish) ancestry group had the lowest estimate at 0.030 individuals per million. In gnomAD version 4.0.0, it was the East Asian ancestry group that had the highest estimated prevalence at 255.23 individuals per million while the European (Finnish) ancestry group similarly had the lowest estimated prevalence at 0.051 individuals per million.

The same set of variants was then used to calculate prevalence using two Bayesian approaches. Both Bayesian approaches yielded similar results to the maximum likelihood calculations (Supporting Information 5).

### 3.4. Calculation 2: Disease Prevalence Including Unreported Likely GNEM-Causing Variants (CADD >23)

Based on our assumption that additional pathogenic variants exist in human populations but have not yet been reported in the literature or variant databases as causing disease, we next performed a calculation including our second set of variants which met our in silico pathogenicity criteria. Accordingly, we focused on the remaining gnomAD variants that lacked clinical associations or were categorized as uncertain significance. This resulted in a list totaling 647 variants. To assess their likely pathogenicity, we followed our described filtering steps which used various in silico tools (Figure 2). Our initial filtration step using MAF reduced the list down to 644 variants. Next, we categorized our 644 variants of unknown significance into three groups: those with a CADD score > 23, of which there were 142, those with a CADD score < 13, of which there were 340 variants, and finally, those variants that did not have an associated CADD score or whose score fell in our defined “Gray Zone” between 13 and 23, of which there were 162 (Supporting Information 3).

With the additional 142 variants whose CADD scores were above 23, we recalculated the disease prevalence using the same calculation methods. This yielded an estimate of 35.99 individuals per million using the maximum likelihood method (Table 2). When including these additional variants, the ancestry groups with the greatest and lowest disease prevalence changed. The African/African American ancestry group had the highest estimate of disease prevalence at 290.74 individuals per million, while the Ashkenazi Jewish ancestry group had the lowest estimate at 0.087 individuals per million. When using both of our updated Bayesian methods, we obtained a similar estimate of prevalence across all ancestry groups (Supporting Information 5).

### 3.5. Calculation 3: Disease Prevalence Including Additional Variants With CADD Scores Above 13 Meeting In Silico Criteria

We subsequently turned our attention to the 162 “Gray Zone” variants and applied additional in silico tools to assess their potential pathogenicity as described in the methods above. Of these 162 variants, only 14 met our additional in silico criteria and were included in an additional estimate of disease prevalence. This group of variants predicted a worldwide disease prevalence of 38.75 individuals per million, with the highest affected ancestry group being African/African Americans with 314.27 individuals per million affected, and the lowest affected ancestry group being the Ashkenazi Jewish ancestry group with only 0.087 individuals per million affected (Table 2). Again, our Bayesian methods showed similar estimates across ancestry groups (Supporting Information 5).

### 3.6. Calculation 4: Disease Prevalence Including Additional Variants With CADD Score Above 13 Irrespective of In Silico Criteria

We finally performed an estimate for disease prevalence using all our “Gray Zone” variants. This method understandably gave a very liberal estimate for disease prevalence with 95.42 individuals per million affected (Table 2). We saw that the South Asian ancestry group had the largest estimate with 480.74 individuals per million affected, while only 0.16 individuals per million were predicted to be affected in the Ashkenazi Jewish ancestry group. Once again, the estimates achieved using the two established Bayesian methods were similar (Supporting Information 5).

These methods gave us four distinct estimates for GNEM disease prevalence ranging from the most conservative at 18.46 individuals per million to the most lenient at 95.42 individuals per million affected according to the established maximum likelihood calculation (gnomAD v2.1.1).

### 3.7. Refinements to Disease Prevalence by Excluding Variant Combinations Unlikely to Result in Individuals Living With GNEM

Lastly, we performed calculations of disease prevalence with additional exclusions to address some of the unique variant combinations in GNEM. As described in the Materials and Methods section, we removed the combination of two null variants appearing together as this would result in embryonic lethality. Additionally, we removed the combination of the Japanese founder mutation p.Asp207Val, the Indian founder mutation p.Val727Met, the common African/African American variant p.Asp239Glu, and the possibility of synonymous variants occurring in homozygosity from being incorporated into the estimate of disease prevalence as these are unlikely to be disease-causing. When accounting for what we termed variant severity, the estimate of worldwide disease prevalence dropped from 18.46 to 11.00 (Table 1). We observed the largest drops in disease prevalence from 201.88 to 19.02 and from 173.91 to 32.32 in the South Asian and African/African American ancestry groups, respectively. These calculations were performed across all our four variant lists (Tables 1 and 2).

After excluding variant combinations unlikely to result in individuals living with the disease, our range of estimates for the global prevalence of GNEM dropped to 11.00–87.68 individuals per million (Tables 1 and 2). Notably, the range was 11.00–28.48 when we limited our inclusion to known pathogenic variants plus variants of uncertain significance with a CADD score greater than 23. Our results show that the previous estimates of GNEM of one to nine in one million individuals are likely an underrepresentation of the true disease prevalence.

## 4. Discussion

Disease prevalence is typically estimated using the number of known individuals with the disease. This approach is inherently flawed, particularly in the field of rare disease, due to the number of individuals who go undiagnosed or misdiagnosed. GNEM poses additional challenges due to its clinical complexity, whereby its presenting symptoms, biochemical, and even histopathological features share considerable overlap with other myopathic disorders [32–34]. Without confirmed genetic testing revealing biallelic *GNE* variants, it is difficult to arrive at a definitive diagnosis of GNEM. For these reasons, current estimates of disease prevalence are assumed to be substantial underestimates and the use of complementary methods that are not based on clinical disease ascertainment is of high value [19]. A previous study based on the results from muscle biopsies at the National Center of Neurology and Psychiatry (NCNP) in Tokyo, Japan, estimated the prevalence of GNEM myopathy to be 0.3 per 100,000 [35]. A similar study based on the results from *GNE* gene screening done in Newcastle, United Kingdom, found that the point prevalence of GNEM in Britain (England, Scotland, and Northern Ireland) was 0.04 per 100,000 [36]. Additionally, work done in Israel and the Middle East using clinical reports and genetic sequencing data has estimated the carrier frequency of the common Middle Eastern founder mutation to range from 1 in 20–25 [3, 37]. Lastly, Celeste et al. used allele frequencies from three exome sequencing databases and estimated the prevalence of GNEM to be roughly 6per million individuals [26].

The goal of this study was to obviate these limitations by estimating the prevalence of GNEM using allele frequencies from population genetic databases. Our most conservative results, using exclusively those variants established in the literature and in variant databases as pathogenic and excluding variant combinations that do not result in individuals living with the disease, provide a prevalence estimate of 11.00 individuals per million. We consider that this is likely to still be an underestimate of true disease prevalence for several reasons. Firstly, this conservative calculation uses only variants reported as pathogenic, and it is reasonable to assume that there are many disease-causing variants that are still unreported. Secondly, even large population databases like gnomAD are not fully representative of global populations when it comes to rare variants, as can be seen from the fact that only 97 of the 376 known pathogenic variants were present in gnomAD v2.1.1. In addition, gnomAD frequencies in specific ancestry subgroups are biased as the database has a strong European predominance due to the greater number of sequencing data available for populations of European ancestry, while other globally important populations are underrepresented. As an example, the Bulgarian Roma founder mutation is only present in one individual in gnomAD v2.1.1 who falls into the “Other” ancestry subgroup. This category has only 6138 exomes compared to the 113,768 exomes covering this location in the European (non-Finnish) ancestry group. Unfortunately, there are no specific ancestry groups for the Middle East or Bulgarian Roma groups in gnomAD v2.1.1, making it difficult to assess the true prevalence of GNEM within these founder populations. With the recent release of gnomAD v4.0.0, which is five times larger than the previous releases and includes over 100,000 individuals of non-European genetic ancestry, there is a jump to a total of 37 individuals with the Bulgarian Roma founder mutation. This release also includes a Middle Eastern ancestry group, allowing us to gain further insight into the prevalence of GNEM within this founder population. The knowledge of these founder mutations demonstrates the importance of not only calculating the overall worldwide population prevalence estimates for GNEM but also breaking down these estimates into ancestry-specific groups.

Additionally, structural variants such as CNVs and large deletions or insertions were not included in our calculations as version 2.1.1 of gnomAD does not report them. Some examples of such GNEM-causing variants include nine unique CNVs reported in an Asian cohort of GNEM patients which most commonly spanned exon 2 of the GNE protein [38]. Other large deletions ranging from single to multiple exons have also been described in the literature and/or reported on ClinVar [12, 24, 39, 40]. The omission of these variants from our calculations of disease prevalence may have resulted in incomplete prevalence estimates. Moreover, our reliance on gnomAD data limited our analysis of both reported pathogenic variants and variants of unknown significance to those present in the gnomAD, potentially missing unreported variants that may be present and/or prevalent in underrepresented ancestry groups in gnomAD. While all our initial calculations and filtering were done using data from gnomAD version 2.1.1, following the release of gnomAD version 4.0.0 during the preparation of this manuscript, we did go back and perform additional calculations on our known variant list using the updated release. The new release of gnomAD increased the number of known pathogenic variants present in the gnomAD (and thus used in our calculation) from 97 to 155, while simultaneously increasing the total number of alleles present in the database. While an increase in the number of variants included in the calculation might be expected to result in an increased estimate for prevalence, these variants are of course divided by a much larger denominator represented by the larger total number of alleles, and as we see, the jump from 141,456 individuals to 807,162 total individuals in version 4.0.0 resulted in a decrease in the worldwide estimate from 18.46 to 11.90 individuals per million.

We made certain assumptions in our estimates of disease prevalence. First, we assumed that variants classified as pathogenic or likely pathogenic in the literature or in genetic databases are correctly classified, which may not always be the case. Additionally, in our analyses of variants of uncertain significance, we assumed that these variants cause GNEM and not other *GNE-*related diseases. In our filtering steps, we relied heavily on CADD scores, using an assumption that those above our designated CADD score cut-off of 23 were pathogenic. Despite the fact that our methodology used CADD scores of known pathogenic variants to determine the cut-offs, this assumption may not be valid. While this manuscript was in preparation, we obtained through personal correspondence a list of mutations from a large cohort of GNEM patients from India, of which 50 were novel variants not appearing in published works or in variant databases. We used these novel variants as proof of principle for our filtering methods by cross-referencing them with gnomAD v2.1.1 and our list of variants of uncertain significance. Of the 50 novel variants, only two (c.1111C>T:p.Gln371^∗^ and c.215T>G:p.Leu72Arg) appeared in gnomAD and had thus been included in our calculations, and both had been classified as likely pathogenic by our methodology as they met our CADD score cut-off of 23. Of the remaining variants, 31 had associated CADD scores, 27 of which met our cut-off of 23. While this is a small sample size, it does support our filtering methods for variants of unknown clinical significance.

Our first set of calculations did not consider certain GNEM-specific features, such as the fact that some variants, like two null mutations, are likely to result in embryonic lethality or that certain variants in homozygosity might not cause penetrant disease. These first calculations thus overestimate the contributions of certain known variants, which prompted us to adjust our calculations to remove these variant possibilities from further estimates. With the incorporation of variant severity in our calculations, we saw a significant decrease in the prevalence estimates particularly in the South Asian and African/African American ancestry groups. The South Asian estimate dropped from 201.88 to 19.02 and the African/African American from 173.91 to 32.32 for the calculation including only known pathogenic GNEM variants. This was not surprising as both the Indian founder mutation p.Val727Met and the common African/African American variant p.Asp239Glu have high minor allele frequencies in gnomAD, and thus, their removal from our calculations when in homozygosity resulted in a drop in these estimates. The overall estimate of disease prevalence also dropped from 18.46 to 11.00. These results demonstrate the importance of accounting for unique variant combinations when performing these types of prevalence calculations, particularly within founder populations.

The detailed breakdown of ancestry groups in gnomAD allows for a more thorough analysis of disease prevalence within these specific groupings, facilitating a deeper assessment of key founder mutations and common variants of interest. However, the uneven representation of different ancestry groups means that such results must be interpreted with caution. Based on our results using data from gnomAD v2.1.1 (Table 1), it is the African/African American ancestry group that has the highest estimated prevalence of GNEM. Notably, this is not reflected in real-life cases of GNEM [41, 42]. There are several potential explanations for this finding, one of which is the lack of emphasis on the diagnosis of GNEM and other rare diseases in African countries [43, 44]. A recent publication suggests the need to emphasize collaborative efforts to better address the presence of rare disease in African populations, which could potentially result in an increase in the described GNEM cases in this population [45]. If this is the case, then we would expect to see an increase in the real-world prevalence of GNEM in African populations as access to next-generation sequencing technologies increases. Another possibility is that the common African/African American variant is not completely penetrant and/or will only cause disease when found in trans with few other strongly deleterious variants. This would result in an exaggerated estimation of disease prevalence within this ancestry group but requires more in-depth analyses on the pathogenicity of this common variant for clarification. Lastly, it is possible that sampling bias in gnomAD, that is, the poor representation of sequencing data from non-European populations, contributes to the inflation of the prevalence estimates in the African and other less well-represented ancestry groups.

Our aim with this study was not to arrive at a single estimate for GNEM disease prevalence, as the limitations that we note above mean that providing such a number would be misleading. Rather, we intentionally provide a wide range of estimates based on different assumptions in terms of the likely clinical significance of unreported variants and inclusion or exclusion of individual variants unlikely to result in individuals living with the disease. We acknowledge the considerable variability in our prevalence estimates, particularly as we incorporate additional variants of uncertain clinical significance. Moreover, the variability is compounded by the utilization of six distinct methods (maximum likelihood with and without variant severity, Bayesian with and without variant severity, and Bayesian with variance with and without variant severity) for calculating disease prevalence. We have used a resampling-based method to estimate 95% confidence intervals for our estimates. It should be noted, however, that those confidence intervals reflect only a small portion of the uncertainty in our estimates, reflecting variability that arises from random sampling of populations where very small proportions harbor any individual variant. They do not reflect uncertainty in which set of variants is correct, how severely they cause disease, nor other factors such as bias in how the population genetics data is collected, or deviations from Hardy–Weinberg equilibria assumptions. As a result, the 95% confidence intervals for different estimates of the same prevalence do not necessarily overlap. Establishing a consistent method for computing confidence intervals across the diverse notions of uncertainty that can affect prevalence estimation poses a considerable challenge beyond the scope of the current study.

Our results also shed light on the utility of the novel AI prediction tools MAVERICK and AlphaMissense. We demonstrate that MAVERICK was more reliably able to predict the pathogenicity of known GNEM variants as compared to AlphaMissense. Nevertheless, the challenges of in silico pathogenicity predictions, in particular for missense variants, would suggest that no tool should be used in isolation, but rather, a suite of tools used in a complementary fashion may provide the most robust method for assessing pathogenicity.

It is striking that our most conservative estimate for GNEM prevalence of 11.00 individuals per million is still substantially higher than previously published estimates of one to nine cases per million. Our most conservative estimate is based only on known pathogenic variants (and indeed only on those known pathogenic variants present in the gnomAD, which represents less than a third of those established by our review) and might thus reasonably be assumed to be an underestimate itself. Previously published estimates were based on clinical ascertainment, which is known to be incomplete. Our estimate is based on variant frequencies in population databases and is thus independent of clinical ascertainment, which makes it a valuable complementary method for estimating prevalence.

A final caveat to our estimates is that as GNEM is an adult-onset disease, individuals carrying disease-causing mutations will not present with a disease phenotype at birth. Thus, our current estimate is an overestimate of the individuals with pathogenic GNEM-causing variants who are currently manifesting an overt phenotype. Based on our most conservative estimate (Table 1—gnomAD v2.1.1 with variant severities) and the current world population of around 8 billion [46], one could assume that approximately 88,000 individuals worldwide have GNEM. However, as approximately half of the world is under the age of 30 [46], the actual present medical burden, that is, individuals with biallelic pathogenic mutations who are seeking medical attention, would then be about half that at approximately 44,000 individuals worldwide.

The establishment of accurate estimates of disease prevalence is critical for increasing diagnostic awareness and for the successful development and design of clinical trials. Currently, genetic testing to identify biallelic mutations in *GNE* remains the gold standard for the diagnosis of GNEM [7]. As awareness of GNEM increases among clinicians and sequencing technology advances and becomes more accessible, we anticipate a rise in the reported cases of GNEM. Ongoing research is also focused on identifying disease-specific blood-based biomarkers, which could further facilitate diagnosis and play pivotal roles in disease monitoring, progression, and response to treatment. Recent years have seen significant progress in understanding the pathomechanism of GNEM, with many promising therapeutics in development [7, 35]. Greater awareness of the disease prevalence and detailed natural history studies will be vital moving forward [47]. In the future, as awareness of GNEM and its prevalence increases, coupled with advancements and improved access to diagnostic techniques and disease monitoring markers, early intervention prior to symptom onset may emerge as a practical and effective approach to treat GNEM.

## 5. Conclusions

Via literature and database review, our study identified 376 pathogenic GNEM variants, which is the most comprehensive list of disease-causing GNEM variants to date. The lack of overlap between the individual databases as well as between the databases and the literature review highlights the challenge of locating reliable pathogenicity information and the importance of obtaining this information from multiple sources.

We developed a novel method of classifying and stratifying likely pathogenic but not yet reported *GNE* variants using in silico tools. We use allele frequencies from the gnomAD population database to provide a comprehensive set of estimates for the worldwide prevalence of GNEM that ranges from 11.00 to 87.68 individuals per million. This exceeds previously published reports of disease prevalence based on clinical ascertainment and highlights the likely underreporting of GNEM. These findings have implications for health economics models, natural history studies, disease burden assessments, and the advancement of drug development and clinical trials.

## Figures and Tables

**Table 1:** Worldwide and ancestry group-specific prevalence estimates using allele frequencies of known pathogenic GNEM variants from gnomAD version 2.1.1 and 4.0.0.

**Ancestry group**	**Maximum likelihood estimate (per million) gnomAD v2.1.1**	**Maximum likelihood estimate (per million) gnomAD v4.0.0**	**Maximum likelihood estimate (per million) gnomAD v2.1.1 accounting for variant severities**	**Maximum likelihood estimate (per million) gnomAD v4.0.0 accounting for variant severities**
All	18.46	11.90	11.00	9.63
African/African American	173.91	204.25	32.32	30.22
Latino/Admixed America	7.54	5.36	6.94	4.02
Ashkenazi Jewish	0.039	8.88	0.039	4.05
Eastern Asian	25.05	255.23	18.09	251.99
South Asian	201.88	179.32	19.02	25.05
Europe (Finnish)	0.030	0.051	0.023	0.051
Europe (non-Finnish)	3.25	3.84	3.16	3.81
Middle Eastern	—	17.83	—	15.97
Other	8.15	—	7.19	—

**Table 2:** Worldwide and ancestry group-specific prevalence estimates using allele frequencies of known pathogenic GNEM variants and variants of uncertain clinical significance according to the maximum likelihood estimate (per million) with and without variant severities.

**Ancestry group**	**Pathogenic GNEM variants + uncertain variants with CADD > 23**	**Pathogenic GNEM variants + uncertain variants with CADD > 23 with variant severity**	**Pathogenic GNEM variants + uncertain variants with CADD > 23 + uncertain variants with CADD > 13 meeting in silico criteria**	**Pathogenic GNEM variants + uncertain variants with CADD > 23 + uncertain variants with CADD > 13 meeting in silico criteria with variant severity**	**Pathogenic GNEM variants + all uncertain variants with CADD > 13**	**Pathogenic GNEM variants + all uncertain variants with CADD > 13 with variant severity**
All	35.99	28.48	38.75	31.05	95.42	87.68
African/African American	290.74	149.12	314.27	172.65	465.08	323.34
Latino/Admixed America	17.08	16.46	18.78	18.17	32.98	32.35
Ashkenazi Jewish	0.087	0.087	0.087	0.087	0.16	0.16
Eastern Asian	29.50	21.97	29.50	21.67	65.50	54.06
South Asian	243.80	60.93	249.97	67.10	480.74	295.22
Europe (Finnish)	0.41	0.41	0.41	0.41	28.92	28.91
Europe (non-Finnish)	13.33	13.16	14.36	14.02	44.99	44.73
Other	17.84	16.88	27.90	26.94	69.03	68.07

## Data Availability

The variants and associated allele frequencies used in our calculations can be found in the supporting information files. The methods used to perform our calculations are detailed in the supporting files, and additional information including the codes can be found at https://github.com/theodorejperkins/Monogenic_Prevalence_with_Severities.

## Supporting Information

Supporting Information 1 Details of the general calculation of disease prevalence accounting for different levels of variant severity as well as a description of the derivation of the confidence intervals.

Supporting Information 2 Table S1: The list of known pathogenic GNEM variants obtained from literature and database sources with associated allele frequencies from GnomAD v2.1.1.

Supporting Information 3 Table S2a–S2c: A list of *GNE* variants of uncertain clinical significance sorted by CADD score. (a) CADD score > 23, (b) CADD score 13–23 meeting additional in silico criteria, and (c) remaining variants with a CADD score > 13.

Supporting Information 4 Table S3: The list of known pathogenic GNEM variants obtained from literature and database sources with associated allele frequencies from gnomAD v4.0.0.

Supporting Information 5 Table S4: A summary table of all of the prevalence estimates using the maximum likelihood with and without variant severity, Bayesian with and without variant severity, and Bayesian with variance with and without variant severity.
