# Supplementary material for: Estimating the Prevalence of GNE Myopathy Using Population Genetic Databases
Source: Hum Mutat. 2024 Aug 29;2024:7377504. doi: 10.1155/2024/7377504 (PMC11919241; doi:10.1155/2024/7377504)
Supplement: Supporting Information 1 — Details of the general calculation of disease prevalence accounting for different levels of variant severity as well as a description of the derivation of the confidence intervals. [file 7377504.f1.docx]

**1. Prevalence estimation with variant severities**

Here we describe the method of calculating the different prevalence estimates for monogenic disease, as well as confidence intervals on those estimates. We derive the estimate equations in the general case that there are variants of three different severities: variants that do not cause disease in homozygosity but that do cause disease when combined heterozygously with a more severe variant (NH for Non-disease-causing-when-Homozygous); variants that do cause disease in homozygosity (DH for Disease-causing-when-Homozygous); and variants that cause embryonic lethality (EL). For readers interested in the traditional autosomal recessive case that all variants cause disease when homozygous, or when combined with each other, then one can simply assume all NH- and EL-related terms in the following equations are zero.

**1.1 Probability of disease**

We assume that each of an individual’s two copies of the disease-causing gene may, independently and randomly, harbor disease-causing variants, according to probabilities that will be defined later. Let V1∈{NH,DH,EL} denote the event that the most severe variant on one copy of the disease gene in an individual is, respectively, NH, DH or EL. And similarly, let V2∈{NH,DH,EL} denote the category of the most severe variant on the individual’s second copy of the gene. Then the appearance of disease after live birth is given by:

Prob( Disease ) = Prob( ( V1=NH and V2∈{DH,EL} ) or

( V1=DH and V2∈{NH,DH,EL} ) or

( V1=EL and V2∈{NH,DH} ) )

In other words, to obtain disease in a live-born individual, we must have pathogenic variants in both copies of the gene, however, the two copies cannot be both NH nor both EL. The former case would not actually cause disease, and the latter case would not result in live birth.

Because of symmetry between V1 and V2, the disease probability can also be written as:

Prob( Disease ) = 2 Prob( V1=NH and V2=DH ) +

2 Prob( V1=NH and V2=EL ) +

2 Prob( V1=DH and V2=EL ) +

Prob( V1=V2=DH)

Now suppose there are N disease-causing variants total, of any of the three types. Let X, Y, and Z denote the variants that fall into the three variant severity classes: X = { i : variant i is NH }, Y = { i : variant i is DH }, and Z = { i : variant i is EL }. Let f_i_ denote the true, but unknown, frequency of variant i within the entire population of interest. Finally, let x, y and z denote the summed variants frequencies in each severity class: x = ∑ _i ∈ X_ f_i_ , y = ∑ _i ∈ Y_ f_i_ , and z = ∑ _i ∈ Z_ f_i_ . Under the usual Hardy-Weinberg equilibrium assumption, and under the assumption that all of x, y, and z are sufficiently small, then the probability of disease is well approximated by:

Prob( Disease ) = 2 x y + 2 x z + 2 y z + y^2^

In the special case that all variants are of the DH type, then this reduces to y^2^, or q^2^ as it was written in the main manuscript.

**1.2 Variant frequencies based on a population genomics database**

We assume the variant frequencies are estimated using a population genomics database. That database reports empirical frequencies of disease-causing alleles out of all alleles observed for each variant position: a variant i∈{ 1, …, N } has been observed C_i_ times out of N_i_ alleles total. We consider two estimates of f_i_ , the first being the maximum likelihood estimate: f_i_^ml^ = C_i_ / N_i_ . The second estimate is Bayesian, and assumes a Beta belief distribution with prior parameters ɑ_i_ and β_i_. Note that the priors are allowed to be specific to the variant, which in our implementation (following previous work) depends on the class of variant, such as missense, nonsense, etc. The code distribution associated with this paper contains a datafile with the prior parameters for each variant type. Naturally, the posterior belief distribution for f_i_ , after accounting for the data in the genomics database, is ɑ_i_’ = ɑ_i_ + C_i_ and β_i_’ = β_i_ + N_i_ - C_i_. The Bayesian philosophy is antithetical to producing a single estimate of a parameter. Nevertheless, the second variant frequency estimate we employ is f_i_^bay^ = E^bay^( f_i_ = ɑ_i_’ / ( ɑ_i_’ + β_i_’ )) = ( ɑ_i_ + C_i_ ) / ( ɑ_i_ + β_i_ + N_i_ ). The expectation E^bay^ in the previous formula is with respect to the posterior Bayesian belief.

**1.3 Estimated probability of disease**

Using the work in the previous section, we can then estimate the probability of disease in three different ways. The first, maximum likelihood estimate is obtained by substituting the maximum likelihood estimates of variant frequencies into the disease probability formula above. That is, if we let x^ml^ = ∑ _i ∈ X_ f_i_^ml^ , y^ml^ = ∑ _i ∈ Y_ f_i_^ml^ , and z^ml^ = ∑ _i ∈ Z_ f_i_^ml^ , then we obtain the estimate:

Prob^ml^( Disease ) = 2 x^ml^ y^ml^ + 2 x^ml^ z^ml^ + 2 y^ml^ z^ml^ + ( y^ml^ )^2^

On the other hand, if we substitute the mean posterior Bayesian estimate for each allele frequency, we obtain our first Bayesian estimate. Let x^bay^ = ∑ _i ∈ X_ f_i_^bay^ , y^bay^ = ∑ _i ∈ Y_ f_i_^bay^ , and z^bay^ = ∑ _i ∈ Z_ f_i_^bay^ , then:

Prob^bay1^( Disease ) = 2 x^bay^ y^bay^ + 2 x^bay^ z^bay^ + 2 y^bay^ z^bay^ + ( y^bay^ )^2^

Our second Bayesian estimate is very similar, but is obtained by taking the expectation, with respect to the Bayesian beliefs, of the full disease probability formula. The only reason this differs from the previous estimate is in the y^2^ term, because of course it is not true that E^bay^( y^2^ ) = (E^bay^ y)^2^. Rather, using V^bay^( y ) = E^bay^( y^2^ ) - (E^bay^ y)^2^ , we have that E^bay^( y^2^ ) = V^bay^( y ) + (E^bay^ y)^2^. Here V^bay^ denotes the variance with respect to the Bayesian beliefs. Therefore our second, or fully Bayesian estimate, for disease prevalence is:

Prob^bay2^( Disease ) = E^bay^( 2 x y + 2 x z + 2 y z + y^2^ )

= 2 x^bay^ y^bay^ + 2 x^bay^ z^bay^ + 2 y^bay^ z^bay^ + ( y^bay^ )^2^ + V^bay^( y )

**1.4 Confidence intervals**

There are many sources of uncertainty in the above estimates, including but not limited to: which variants are pathogenic, what their severities are, whether the Hardy-Weinberg equilibrium assumption is reasonable, whether maximum likelihood estimates or Bayesian estimates are reasonable, and whether the population database frequencies are accurate representations of the true population frequencies of the variants. It is far beyond the scope of this study to account for all of these sources of uncertainty. Here we describe a simple resampling method that aims to capture only a portion of the uncertainty in our estimates having to do with how well observed allele frequencies match true population frequencies. Even this method does not account for the possibility that alleles with zero observed frequency may not have zero frequency in the population.

We perform K times a random resampling of the C_i_ values in the population database by independently randomly generating binomial random variables with success probability p_i_ = C_i_ / N_i_ and N_i_ tries. Based on the resampled C_i_ values we calculate the probability of disease according to one of the three formulas above. We then sort these values from smallest to largest and take the boundaries of the middle 95% of these values as our confidence interval. In our current software implementation, K = 1000, and we take the 25th and 975th largest values for the ends of the confidence interval.
